# Investigation of Distribution of Heavy Metals between Blood Plasma and Blood Cells

**DOI:** 10.1002/adic.200790118

**Published:** 2007-11

**Authors:** Stasys Tautkus, Algimantas Irnius, Danute Speiciene, Jurgis Barkauskas, Aivaras Kareiva

**Affiliations:** Department of Analytical and Environmental Chemistry, Vilnius UniversityLT-03225 Vilnius, Lithuania; Centre of Gastroenterology and Dietetics, Vilnius UniversityLT-08661 Vilnius, Lithuania; Department of General and Inorganic Chemistry, Vilnius UniversityLT-03225 Vilnius, Lithuania

## INTRODUCTION

The physiological importance of metals in human organism has been shown by many publications. The toxic doses of metals and their compounds can lead to serious health problems.^1^–^4^ Different metals present in the composition of blood can form different complexes with many organic compounds and biomolecules which could be found in the body fluids.^5^–^7^ Depending on the concentration of metals in the parts of body, different metal-ligand equilibriums could be established in the system. These changes could cause changes in global bioprocesses, or different clinical symptoms and metabolic stresses in human organism could occur. The distribution of metals between blood plasma and blood cells could represent an important clinical index.^8^–^10^ The aim of the present study was to investigate, for the first time to our knowledge, the distribution of heavy metals between blood plasma and cells in the blood samples from the infected by *hepatitis C* and non-infected patients.

## EXPERIMENTAL

The amount of metals in the blood samples has been determined by flame atomic absorption spectroscopic method (FAAS, Hitachi 170–50). The instrumental parameters were adjusted according to manufacturer's recommendations. For the determination of the total amount of metals in blood samples, the specimens were burnt in the ordinary furnace at 800°C. The obtained residuals were dissolved in 10 mL of nitric acid (1:1), transferred into a 25–mL volumetric flask, and diluted with doubly-distilled water. For the analysis of the metal concentration distributions between blood plasma and blood cells, separation of plasma from cells was performed by centrifugation (spin speed 8000 min−^1^) prior to FAAS determination. Full separation of plasma from cells was achieved after 6–8 minutes. The blood samples were taken under informed consent from 36 volunteer patients infected by *hepatitis C*. For the comparison, the 36 blood samples from noninfected patients were also analyzed.

## RESULTS AND DISCUSSION

The results for the determination of the total amount of 9 metals in the plasma and blood cells from the infected and non-infected patients are summarized in Tables 1 and 2.

**TABLE 1:** Results obtained for the determination of total amount of metals in blood plasma from infected by hepatitis C and non-infected patients.

	Blood plasma			
Metal	Infected by hepatitis C (36 samples)		Non–infected by hepatitis C (36 samples)	
	Amount of metal, μg/g^a^	R.S.D., %	Amount of metal, μg/g^a^	R.S.D., %
Cr	-		0.28	12.1
Mn	0.15	10.1	-	
Fe	3.5	8.8	2.13	10.8
Co	-		-	
Ni	-		-	
Cu	3.49	11.3	0.81	9.0
Zn	7.44	10.2	7.73	9.9
Cd	-		-	
Pb	-		-	

a Average of five independent determinations

**TABLE II:** Results obtained for the determination of total amount of metals in blood cells from infected by hepatitis C and non-infected patients.

	Blood cells			
Metal	Infected by hepatitis C (36 samples)		Non-infected by hepatitis C (36 samples)	
	Amount of metal, μg/g^a^	R.S.D., %	Amount of metal, μg/g^a^	R.S.D., %
Cr	1.21	8.9	1.48	11.9
Mn	0.09	10.6	0.29	11.4
Fe	599.5	10.0	604.3	8.3
Co	-		-	
Ni	-		-	
Cu	3.55	10.1	1.22	9.7
Zn	7.62	11.8	7.47	11.6
Cd	-		-	
Pb	0.17	9.3	0.77	10.9

a Average of five independent determinations

It is interesting to note that, according to FAAS analysis data, Co, Ni and Cd are present neither in the infected nor in non–infected blood samples, or their concentrations are lower than the detection limit determined for these three elements. As expected, the concentration of Fe is much higher in comparison with other elements. The concentrations of the biologically important elements Cu and Zn were found to be significantly higher than the Cr and Mn concentrations. In general, looking to each patient the blood samples contain more Zn than Cu. The Cr content found in the specimens is higher in comparison with Mn content in all of the cases.

From the results presented in Tables 1 and 2 we can conclude that, as expected, Fe predominates in blood cells, and only small amount remains in blood plasma. However, we have observed that the distribution of Fe between blood cells and plasma seems to be independent on the infection. Absolutely no difference in the distribution of Cu between plasma and blood cells was detected. A very similar situation was found also in the case of distribution of Zn. Therefore, we can conclude that Cu and Zn can form in the blood a variety of different complexes which have different affinity to plasma and cells. Interestingly, blood cells infected by *hepatitis C* contain a much higher concentration of Cu in comparison with non-infected samples. On the other hand, Zn is distributed almost evenly between blood cells and plasma in both cases.

A random distribution of Cr and Mn in plasma and cells is also evident from the results presented in Tables 1 and 2. However, these elements show slightly different distribution between plasma and blood cells. For example, after centrifugation all Cr remained in blood cells only for the infected blood samples. On the other hand, Mn remained in blood cells only for the blood samples from non–infected patients. Finally, during the separation procedure all Pb remains in blood cells regardless the blood sample.

In conclusion, these results indicate that metals exist in blood in different physico–chemical forms. These preliminary observations give indication that the concentration of Cu, Cr and Mn in blood cells and in plasma could be a signal of the seriousness and depth of the disease. Concentrations of the other three elements (Fe, Zn and Pb) do not vary significantly between blood cells and plasma in infected or non-infected patients. The results show various distributions of different metals in blood plasma and blood cells. This could be explained only by the existence of particular physico–chemical forms of metals in blood. Since these species probably possess different affinity to plasma or cells, possible different accumulation of certain metals in blood cells and plasma could be achieved. Different accumulations might be the sign or the possible reason of appearance of symptoms of some disease. Of course, such a correlation could be made only after a careful and systematic medical investigation of a large number of patients.

## References

[1] Zorbas YG, Federenko YF, Naexu KA (1994). Biol. Trace Elem. Res.

[2] Tashiro H, Okubo T (2002). Biomed. Res. Trace Elem.

[3] Kindness A, Sekaran CN, Feldmann J (2003). Clinic. Chem.

[4] Ozcelik D, Toplan S, Akyolcu MC (2004). Trace Elem. Electrol.

[5] Pagani S, Mirtella D, Mencarelli R, Rodriguez D, Cingolani M (2005). J. Anal. Toxicol.

[6] Hackett J (2006). J. Anal. Toxicol.

[7] Day D, Kuntz DJ, Feldman M, Presley L (2006). J. Anal. Toxicol.

[8] Dunne AL, Mitchell F, Allen KM, Baker HWG, Garland S, Clarke GN, Mijch A, Crowe SM (2003). J. Clin. Virol.

[9] Tuschl K, Bodamer OA, Erwa W, Mühl A (2005). Clin. Chim. Acta.

[10] Golec E, Suszko R, Nowak S, Piatkowski M, Chrzanowski R, Schlegel-Zawadzka M, Gozdzialski R (2005). Trace Elem. Electrol.

[11] Maurer HH (2005). Anal. Bioanal. Chem.

[12] Hayashi N, Takehara T (2006). J. Gastroenterol.

